# The impact of interventions to reduce risk and incidence of intimate partner violence and sexual violence in conflict and post-conflict states and other humanitarian crises in low and middle income countries: a systematic review

**DOI:** 10.1186/s13031-021-00417-x

**Published:** 2021-11-24

**Authors:** Jo Spangaro, Chye Toole-Anstey, Catherine L. MacPhail, Delia C. Rambaldini-Gooding, Lynne Keevers, Claudia Garcia-Moreno

**Affiliations:** 1 Faculty of the Arts, Social Sciences and Humanities, School of Health and Society, University of Wollongong, Northfields Avenue, Wollongong, NSW 2522 Australia; 2 Department of Sexual and Reproductive Health and Research, World Health Organization, Avenue Appia 20 – 1211, Geneva, Switzerland

**Keywords:** Systematic review, Sexual violence, Sexual assault, Domestic violence, Intimate partner violence, Gender-based violence, Armed conflict, Humanitarian crisis, Outcomes

## Abstract

Sexual violence and intimate partner violence are exacerbated by armed conflict and other humanitarian crises. This narrative systematic review of evidence for interventions to reduce risk and incidence of sexual and intimate partner violence in conflict, post-conflict and other humanitarian crises, updates and expands our review published in 2013. A search of ten bibliographic databases for publications from January 2011 to May 2020 used database specific key words for sexual/intimate partner violence and conflict/humanitarian crisis. The 18 papers, describing 16 studies were undertaken in conflict/post-conflict settings in 12 countries. Six intervention types were reported: i) personnel; ii) community mobilisation; iii) social norms; iv) economic empowerment; v) empowerment; and vi) survivor responses, with the most common being economic empowerment (n = 7) and gendered social norms interventions (n = 6). Combined interventions were reported in nine papers. Four studies identified non-significant reductions in incidence of sexual/ intimate partner violence, showing an evident positive trend; all four evaluated gendered social norms or economic empowerment singly or in combination. Evidence for improved mental health outcomes was found for some economic empowerment, social norms and survivor interventions. Some evidence of reduced *risk* of sexual violence and intimate partner violence was identified for all intervention types. Qualitative studies suggest that experiences of social connection are important for women who participate in programming to address sexual and intimate partner violence. Interventions with multiple strategies appear to hold merit. Achieving and demonstrating reduced sexual and intimate partner violence remains challenging in this context. Future research should continue to explore how social norms interventions can be most effectively delivered, including the impact of including mixed and same sex groups. Work is needed with local partners to ensure programs are contextually adapted.

**Supplementary Information:**

The online version contains supplementary material available at 10.1186/s13031-021-00417-x.

## Introduction

Gender-based violence, prevalent during peace, is exacerbated in armed conflict and other humanitarian crises [1–4]. This review focuses specifically on sexual violence and intimate partner violence, two most prevalent forms of gender-based violence. Sexual violence is defined as “any sexual act, attempt to obtain a sexual act, unwanted sexual comments or advances, or acts to traffic, or otherwise directed against a person’s sexuality, using coercion, by any person, regardless of their relationship to the victim, in any setting, including, but not limited to, home and work” [5] (p. 149). Intimate partner violence refers to “a pattern of behaviour by a current or former partner causing physical, sexual or psychological harm, such as physical aggression, sexual coercion, psychological abuse and/or controlling behaviours.”[6] (p. vii).

Available estimates for prevalence of sexual violence among women in humanitarian emergencies range from 21 to 53% [7–9] and for intimate partner violence 12–80%, [10–14] although prevalence in this context are considered to be under-reported. [15–17] Forms of sexual violence are perpetrated by armed actors, aid workers, peacekeepers, intimate partners, family and community members. Sexual violence and intimate partner violence can result in serious long term physical and mental health effects [18, 19], including adverse pregnancy outcomes, maternal mortality, and sexually transmitted infections, as well as heightened risk of stigma, isolation, stress, reduced ability to negotiate sex, forced pregnancy, and unsafe sexual practices [7, 10–12, 20–23].

Factors exacerbating sexual violence and intimate partner violence in crises include: exposure to armed conflict; increased availability of weapons; perceived threats to masculinity; increased substance abuse; human rights abuses; extreme poverty; loss of livelihoods; disrupted family and community protection structures; traumatic stress; loss of legal and protective mechanisms; disability; pre-existing intimate partner violence; transactional sex, and lost access to basic resources [1, 9–11, 20, 21, 23–30].

### Background

This systematic review updates and expands one published in 2013 on evidence for interventions to reduce incidence and risk of sexual violence in contexts of conflict, post-conflict and other humanitarian crises [31, 32]. That review found some evidence of decreased sexual violence in association with firewood distribution and from programming to reduce sexual exploitation by peacekeepers. Apparent increased risk occurred through lack of protection, stigma and retaliation associated with interventions. Significant barriers were identified which prevented women seeking help after sexual violence, although multiple component interventions and sensitive community engagement appeared to contribute to positive outcomes [31].

Documentation of intimate partner violence in humanitarian contexts has shown that it is as, or more, prevalent than sexual violence [15] which has led to the increased recognition of the need to address intimate partner violence and other forms of gender-based violence in these settings. Furthermore, the call to address the social and economic drivers of gender-based violence in emergencies have expanded the focus beyond survivor response to also include primary prevention [16]. Initiatives driving this work include the ‘IASC Guidelines for Integrating Gender-Based Violence Interventions in Humanitarian Action’ outlining actions for mitigating risk of and preventing gender-based violence [33], as well as the What Works? Research consortium-seeking evidence on preventing violence against women and girls, including in conflict and humanitarian crises. Further appraisal of the evidence has occurred through systematic reviews of programming for gender-based violence in refugee settings [4, 34] and of psychosocial support interventions in conflict zones [24, 35]. Post-settlement programs are not included, given the different context in which these are provided and accordingly, scope is restricted to low and middle income countries. This review examines trends in prevention and reduction of risk of sexual violence and intimate partner violence across conflict, post–conflict and other humanitarian settings, a current gap in the literature.

## Methodology

This review sought to answer the question: What is the evidence of the impact of programmes/interventions to reduce risk and incidence of intimate partner violence and sexual violence in conflict and post-conflict states and other humanitarian crises in low and middle income countries? While recognising that other forms of gender-based violence are prevalent, given the wide scope of this review we excluded interventions which address: trafficking and female genital mutilation; directed towards children, apart from those related to preventing early, child and forced marriage, or where these are part of interventions aimed at intimate partner violence and sexual violence. This review recognises men may be affected by sexual violence especially during conflict, however focused on abuse directed towards women, given higher risk and prevalence for women [6, 13, 15]. The review is registered with PROSPERO, CRD42020186405.

### Conceptual framework

Our approach built on the conceptual framework applied in the 2013 review, updated based on advice from the WHO and an expert panel convened by the WHO. Drawing on an ecological approach, interventions were conceptualized as operating at the level of individual, family, community or society [36] (Table 1) recognising that multi-strategy programs which span different levels are increasingly delivered in humanitarian contexts.

**Table 1:** Intervention and strategy type

Intervention type	Strategies and examples
Society	*Personnel* use of code of conduct training and policies to reduce opportunity by personnel for sexual exploitation and abuse; deployment or increased recruitment of female officers
	*Peace building for prevention of sexual violence* incorporation of sexual prevention measures in ceasefire negotiations and monitoring
	*Strengthening the legal and regulatory environment* law and policy reform to criminalise and strengthen protections against violence against women
	*International, national and customary justice mechanisms*
Community	*Social norms interventions* strategies that aim to transform harmful gender norms and stereotypes that justify violence against women
	*Community mobilisation* engagement of community leaders, institutions and members to take action to address and prevent violence against women
	*Infrastructure* risk mitigation efforts to create safe spaces
	*Security* provision of foot and vehicle patrols/security details to vulnerable areas; establishment of safety protocols
	*Reduced availability of alcohol/ other drugs* bans/ curfews on trading, declaration of dry areas or communities
Family	*Relationship skills programs* strategies aimed at individuals or groups of women, men or couples to improve skills in interpersonal communication, conflict management and shared decision making
	*Educational programs* to build skills, attitudes and expectations for respectful relationships
Individual	*Survivor response* provision of care, support and protection to assist survivors to heal, recover and reduce further exposure to violence
	*Perpetrator education/intervention* aimed at men who commit acts of sexual / intimate partner violence
	*Empowerment* strategies to build women’s skills in assertiveness, negotiation, and self-confidence
	*Economic empowerment* provision of money and/or training and/or support to women to increase their economic independence
	*Combatant-focused interventions* including Disarmament, Demobilization and Reintegration programs targeting reduced SV engaging with combatants & leaders

Reducing incidence of sexual violence and intimate partner violence is a long-term outcome, which is difficult to achieve and measure accurately in any context, but particularly in crisis settings. Reduced risk is a key intermediate outcome. A non-exhaustive list of indicators adapted from *Violence Against Women and Girls: A Compendium of Monitoring and Evaluation Indicators* (Bloom 2010) used in the 2013 review, was updated to reflect inclusion of intimate partner violence and to incorporate elements of the RESPECT Women framework which reflects current approaches to gender based violence prevention [37] (Additional file 1). Inclusion and exclusion criteria (Table 2) were determined with advice from the expert panel. We included interventions delivered to refugee/migrant women in Greece, although it is not a LMIC, given the large number of refugee arrivals directly to this country.

**Table 2:** Inclusion and exclusion criteria

	Included	Excluded
1. Focus	Intimate partner violence (IPV) and/or sexual violence (SV) against women/girls in the context of conflict or humanitarian emergency—including forced marriage/pregnancy, sexual slavery, enforced sterilization. IPV includes violence in the context of child marriage	Studies not addressing SV/IPV. Studies addressing female genital mutilation, trafficking, or HIV prevention, except where interventions to address these issues are part of interventions aimed at SV/ IPV, SV within military organizations. Interventions for violence directed towards children apart from adolescents or violence in the context of child marriage
2. Studies/data	Empirical studies describing the outcomes of interventions	Studies describing nature/extent/ impacts of SV/IPV or barriers to implementation of, or interventions not specific to SV/IPV; papers lacking primary empirical data describing impacts of interventions
3. Participants	Participants in interventions to address SV/ IPV including survivors, service providers, perpetrators, male/female community members, institution representatives, armed groups or humanitarian workers	Commentators or actors not directly involved in implementation or experience of interventions
4. Interventions	Interventions aimed at reducing the incidence of or risk of SV/ IPV and other forms of GBV where these are also targeted by the intervention, or interventions where reduced risk of SV/IPV was a measured outcome of the intervention	Interventions that do not refer to reduction of SV/IPV as a specific aim or outcome e.g. peace-building /community development programs or interventions aimed at HIV prevention
5. Settings	Contexts of conflict, post-conflict or other humanitarian, emergency, including epidemic outbreaks in low and middle-income countries as defined by the World Bank 2019	Context of the intervention not identified by the author(s) in title/abstract as conflict/post-conflict or humanitarian crisis. Interventions not conducted in the context of conflict/post-conflict or humanitarian crisis. Countries not listed as LMIC by the World Bank Note: Interventions delivered in Greece to refugees/migrants from LMICS considered for inclusion
6. Publications	Research papers or research reports	Letters, editorials, comment, periodicals, editorials, art works, news updates, speeches
7. Language of publication	English	Titles and abstracts in a language other than English
8. Publication date	September 2011–May 2020	Before September 2011 (end date of search in 2011 review)

### Searches

**Fig. 1:**
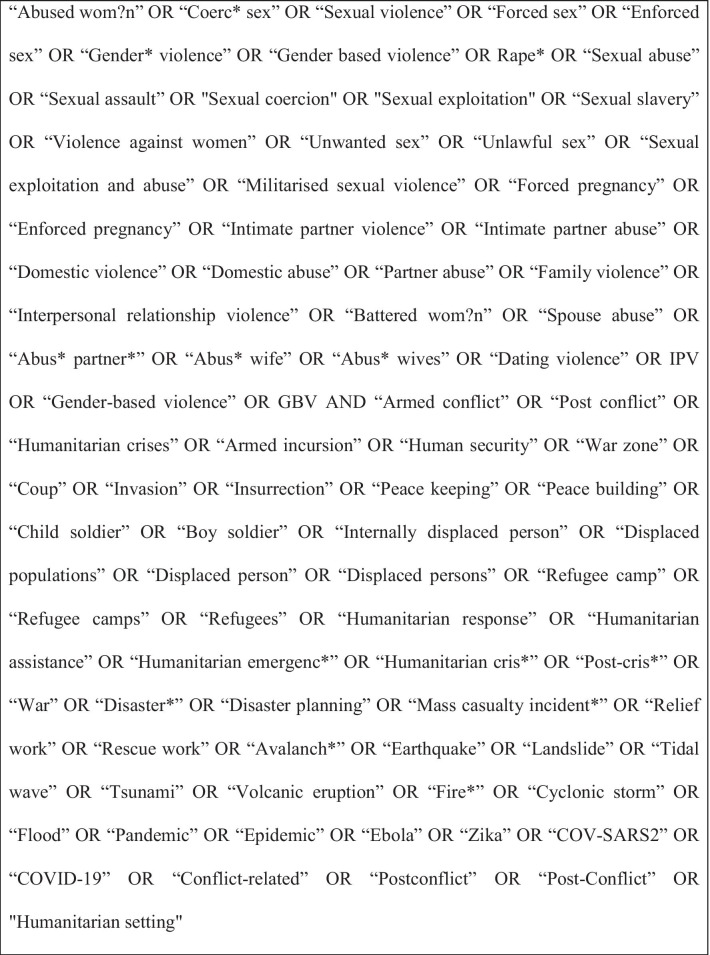
Sample search strategy (Psycinfo)

Searches were conducted in May 2020 on the following databases: CINAHL; Cochrane Library; EMBASE; GlobalHealth; Johanna Briggs Institute EBP; Proquest; PTSDPubs; PSYCInfo; Pubmed Central; Scopus. Search terms for “sexual violence” or “intimate partner violence” and “conflict” or “post-conflict” or “humanitarian crisis” and their synonyms were used to search key terms/subject, title and abstract. The search of the PsycINFO database is reported in Fig. 1 as an example of the approach used. Grey literature was hand-searched via: Institutional Repository for Information Sharing, Google Advance and Campbell Collaboration database using the same search terms.

### Screening and quality review

The title and abstracts, and then full-text were screened by pairs of team members independently. Divergent results were resolved by the team as a whole. The Johanna Briggs Institute (JBI) Quality Appraisal Checklists (http://joannabriggs-webdev.org/research/critical-appraisal-tools.html were applied to assess quality of papers, selected as these tools allow for diverse study types to be assessed. These tools assess the methodological quality including the risk of bias which may be present in research design and analysis. Team members in pairs double assessed individual studies, (LK, CM, DRG, CTA) with one team member (JS) reviewing the overall results. Quality was not used to exclude studies.

### Data extraction and analysis

Using a standardized template, data were extracted and coded by two team members on: country of intervention; strategy type; intervention; target population; duration and intensity of intervention; study design and participants; organization type; context; reported outcomes and unintended consequences. Studies were also rated for decrease/increase or no change to risk or incidence of sexual violence/intimate partner violence. Data were analysed as a team using narrative synthesis [38], involving close reading of each text and consideration of each strategy type against the review questions: evidence for i) reduced incidence of sexual violence/intimate partner violence; ii) reduced risk of sexual violence/intimate partner violence as a result of interventions, including identification of new indicators as agreed by the team; and iii) impacts of secondary prevention interventions on survivors’ wellbeing. Qualitative findings were used to amplify and contextualize results from quantitative studies.

## Results

**Fig. 2:**
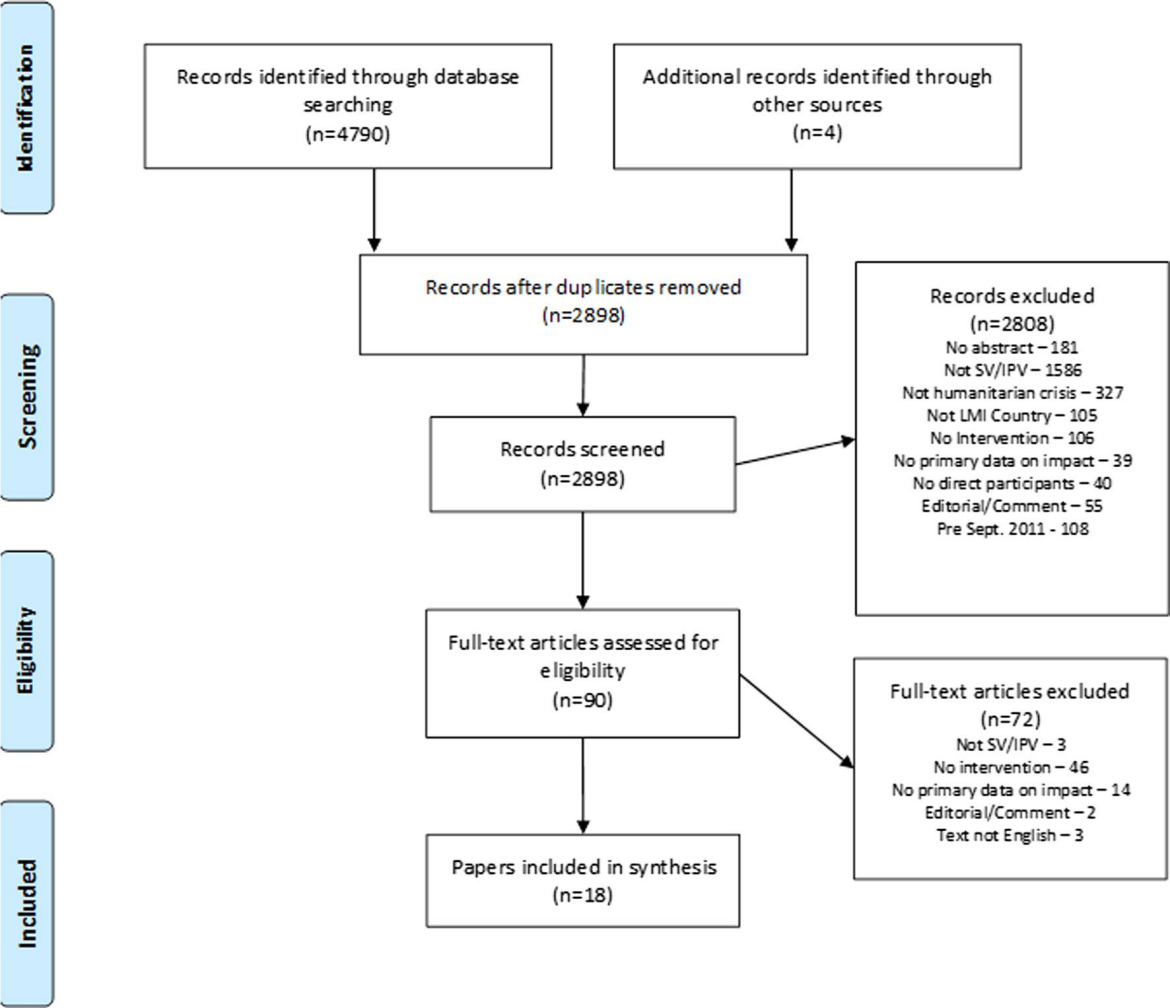
Search strategy and filtering

The search strategy yielded 4794 records reduced to 2898 after de-duplication. Screening for title and abstract yielded 90 titles, reduced to 18 papers following full text review (Fig. 2). Only one inclusion was identified through the grey literature search [39].

### Studies identified

Eighteen articles were included in the review, reporting results from 16 separate studies, summarised in Table 3. Articles by Gupta, Falb et al. [40], Falb, Annan et al. [41] and Annan, Falb et al. [42] all reported on a single intervention addressing economic empowerment and social norms as denoted by * in Table 3. Interventions were conducted in 12 countries, with the greatest number (n = 5) occurring in the Democratic Republic of Congo (DRC). Nine studies were conducted in post-conflict, five in conflict and two in combined conflict and post-conflict settings. All humanitarian crises were in scope, however no studies which met the inclusion criteria were identified from disaster or epidemic settings. Seven of the 16 included studies targeted intimate partner violence, seven targeted both sexual violence and intimate partner violence, while two addressed sexual violence only.

**Table 3:** Summary of included papers

Author (s)	Country	Strategy type	Abuse type & population	Name & intervention	Study type
Annan et al. * [42]	Cote D'lvoire	Economic empowerment + Social norms	IPV − Women (18 yrs +) & partners	EA$E − Group savings + 8 × 1.5–2.5 h gender dialogue groups compared to group savings only	RCT
Bacon [48]	Liberia	Personnel	IPV − Female police recruits	Recruitment and training + creation of specialist unit	Case Study
Bass et al. [43]	DRC	Survivor response	SV − Women survivors of SV with PTSD/anxiety + depression	12 × 2 h group sessions cognitive processing therapy compared to individual support only	CRCT
Falb et al. * [41]	Cote d'Ivoire	Economic empowerment + Social norms	IPV − Women (18 yrs +) & partners	EA$E − Group savings + 8 × 1.5–2.5 h gender dialogue groups compared to group savings only	Qualitative- Interview
Gibbs et al. [52]	Afghanistan	Economic empowerment + Social norms	IPV − Women (18–49 yrs) identified with social and economic vulnerability	WfWI − 2 × 1.5–3 h weekly × 12 months + empowerment and vocational skills + cash transfer + group savings	Mixed Methods
Glass et al. [46]	DRC	Economic empowerment	IPV − Women and men community members (16 yrs +)	Pigs for Peace − Microcredit/livestock product asset transfer program compared to wait list	RCT
Glass et al. [44]	Somalia	Social norms	SV + IPV − Women and men community members (15 yrs +)	Communities Care − Weekly discussion groups × 15 compared to wait list	CRCT
Green et al. [49]	Uganda	Economic empowerment + social norms	IPV − Low income earning women and their partners	WINGS − 5 days business training; start up grant; 6 weekly follow up visits for 6 months compared to delayed treatment. Compared to WINGS + or Women Plus variant	CRCT
Gupta et al. * [40]	Cote d'Ivoire	Economic empowerment + social norms	IPV − Women (18 yrs +) & partners	EA$E − Group savings + 8 × 1.5–2.5 h gender dialogue groups compared to group savings only	RCT
Gurman et al. [45]	South Sudan, Uganda, Rwanda, Thailand, Liberia	Community mobilisation	SV + IPV − Community members & NGO workers	Through Our Eyes − Community awareness participatory video project. Videos approx. 20 min long with group session x 60 min	Qualitative- Interview + FGDs
Hossain et al. [51]	Cote d'Ivoire	Social norms	IPV − Men -community members (15 yrs +)	Men & Women in Partnership initiative − Weekly male discussion group sessions × 16 compared to community-level programming for raised awareness on women’s rights and GBV impacts	CRCT
Hossain et al. [39]	Kenya	Survivor response + community mobilisation	IPV − Women refugees (15 yrs +) after IPV/SV	Case management + psychosocial support + GBV messaging	Mixed Methods
Koegler et al. [47]	DRC	Economic empowerment	SV − Women − survivors of conflict-related SV	Group credit and animal asset transfer; shared farming; weekly group support meetings − Solidarity Groups 1–2 h	Qualitative − Interview
Lilleston et al. [54]	Lebanon	Survivor response + community mobilisation	SV + IPV − Refugee women and adolescent girls	GBV mobile service delivery approach − 6 months of weekly individual case management; psychosocial support; information sessions; door-to-door visits	Qualitative − Interview
Stark et al. [55]	Ethiopia	Empowerment + relationship skills	SV + IPV − Girls (13–19 yrs) and their mothers	COMPASS − 30 life skills sessions × 1–3 h + 8 mothers’ sessions (length not reported) compared to wait list	CRCT
Stark et al. [56]	DRC	Empowerment + relationship skills	SV + IPV − Girls (10–14 yrs) and their mothers	COMPASS − 32 life skills sessions × 1-3 h + 13 mothers sessions (length not reported) compared to wait list	CRCT
Vaillant [50]	DRC	Social norms	IPV − Men community members (18 yrs +)	EMAP − 16 × weekly 3 h male discussion group sessions compared to non-gender norms-related alternative group sessions with discussion topics chosen by group members	CRCT
Vu et al. [57]	Kenya	Survivor response	SV + IPV − Women refugees (15 yrs +)	Health clinic screening for SV + IPV	Mixed Methods

*Derives from the EA$E study (three papers)

Of the 16 possible intervention types identified in the conceptual framework, (Table 1) evidence was found for six: i) personnel; ii) community mobilisation; iii) social norms; iv) economic empowerment; v) empowerment and; vi) survivor responses. Almost half (n = 7) of the 16 studies combined two intervention types. Duration and intensity of the intervention is indicated in Table 3 for all studies where this was reported. As reported in papers, ten of the interventions were delivered by international non-government organizations (NGOs). Three were delivered jointly by international and local NGOs [42–45]. Two interventions were implemented by local NGOs alone [46, 47] and one by a state agency [48]. Data were not available for most studies on length of implementation prior to measuring outcomes, with nine run specifically as trials.

Table 4 summarises the evidence for reduced risk and changed incidence of intimate partner violence and/or sexual violence as well as the key impacts of the interventions. Four studies identified reductions in incidence of intimate partner violence [40, 46, 49, 50] and one of sexual and intimate partner violence [51], though none reached statistical significance. One showed reduced intimate partner violence only among a sub-set of women with moderate baseline food insecurity [52]. These six studies evaluated social norms [50, 51], or economic empowerment interventions [46] or both combined [40, 49, 52]. Some evidence of a reduction in factors associated with *risk* of sexual violence/intimate partner violence was identified in all studies against the identified list of indicators, to which we added five further indicators during the analysis. These were: household poverty reduced; increased economic autonomy for women; changed norms supporting women’s equality; changed norms on unacceptability of gender-based violence in men. In addition, strong evidence was present for projects that focused on economic empowerment [46] and economic empowerment plus social norms [42] for improved mental health outcomes.

**Table 4:** Changes to risk, incidence and key effects of included studies

Author (s)	Change to incidence	Change to risk	Violence and context	Indicators for reduced risk	Key impacts
Personnel					
Bacon [48]	Not applicable (NA)	↓	IPV PostC	Gender specific recruitment implemented Implementation/impacts of codes of conduct/training	Increased GBV reporting to police. Enhanced awareness of and response to GBV. Gaps include capacity building and training, and officer attrition. Few cases proceeded to court. Weak justice system reduced effectiveness
Community mobilisation					
*Gurman *et al*. *[45]	*NA*	*↓*	*SV* + *IPV* *PostC*	*∙∙Awareness of rights by community* *∙∙Willingness to use reporting mechanisms*	*Changes in family gender dynamics. Improved communication within the family. Women suggested reduced violence* + *greater freedom. Males reported increased knowledge about GBV* + *changed behaviour. Reports of persisting culture of silence*
Social norms					
Glass et al. [44]	NA	↓	SV + IPV PostC	∙∙Changed norms on acceptability of VAW ∙∙Acceptability of services/reporting mechanism	Male and female participants showed significant positive change to social norms on all 3 subscales: blame for SV (b = − 0.214, *p* = 0.041); protecting family honour(b = − 0.558, *p* < 0.001); husband’s right to use violence (b = − 0.309, *p* = 0.003)
Hossain et al. [51]	↓ Not significant (NS)*	↓	SV + IPV C	∙∙Changed norms supporting women’s equality ∙∙Changed norms on acceptability of VAW ∙ Men show reduced acceptance of VAW	At 12 months reduced SV + IPV (aRR 0.52, 95% CI 0.18–1.51 (NS). Also NS reports by men of reduced intention re physical IPV (aRR 0.83, 95%CI 0.66–1.06) and increase in support for women’s right to refuse sex (aRR 1.21, 95%CI 0.77–1.91). Men’s reported involvement in household tasks (aRR 2.47,95% CI 1.24–4.90) and ability to control hostility increased (aRR 1.3,95% CI 1.06–1.58)
Vaillant et al. [50]	↓NS	↓	IPV PostC	∙∙Changed norms supporting women’s equality ∙∙Men show reduced acceptance of VAW	At 12 months NS differences in IPV (aOR = 0.95; SE = 0.14; *p* = 0.71) but women perceived reduced ‘negative male behaviour’ (β = − 0.32; *p* < 0.01). Males reported decreased agreement with wife beating. (OR = 0.59; SE = 0.08; *p* < 0.01) + increased agreement with right to refuse sex (OR = 1.47; SE = 0.24; *p* < 0.05)
Economic empowerment					
Glass et al. 2017 [46]	↓ (NS)	↓	IPV PostC	∙∙Increased economic autonomy ∙∙Household poverty reduced ∙∙Improved survivor wellbeing/mental health	At 18 months reduced psychological abuse (*p* = 0.80) (NS), increased livestock/animal assets (p = 0.00004) + reduced debt (p = 0.028) + improved subjective health (p = 0.035), anxiety (p = 0.023) and PTSD (p = 0.0004)
*Koegler *et al*. 2019 *[47]	*NA*	*↓*	*SV* *C*	*∙∙Increased economic autonomy*	*Women described improved mental health and increased connection and security from shared farming and improved relationships in the community*
ECONOMIC EMPOWERMENT + SOCIAL NORMS					
Annan et al. [42]	Not reported (NR)	↓	IPV C	∙∙Increased economic autonomy ∙∙Changed norms supporting women’s equality	Gender dialogue group plus group savings—significantly less likely to meet PTSD criteria than group savings alone (OR: 0.61; 95% CI: 0.40–0.93; *p* = 0.02) but non-significant among partnered women who had experienced IPV at baseline (OR: 0.72; 95% CI: 0.29–1.82; *p* = 0.5)
*Falb *et al*. 2014 *[41]	*NA*	*↓*	*IPV* *C*	*∙∙Changed norms supporting women’s equality* *∙∙Changed norms on acceptability of VAW*	*Most male participants were positive about women’s participation in group savings with financial benefits as primary motive. Participants described increased joint decision making, reduced arguments and “violent problem solving.”*
Gibbs et al. [52]	↓ (sub-group)	↓	IPV	∙∙Increased economic autonomy ∙∙Household poverty reduced ∙∙Changed norms supporting women’s equality	Women with moderate food insecurity at baseline showed decreased past year physical IPV and severe physical IPV. For all, higher mean earnings and savings at the end of the program. Increased female autonomy and economic empowerment beneficial but limited impact due to extreme poverty and patriarchal social norms
Green et al. [49]	↓ (NS)	↓	IPV Post-C	∙∙Increased economic autonomy ∙∙Changed norms supporting women’s equality	Inclusion of partner had little impact on economic outcomes compared to economic empowerment only.Small decline in marital control (β = 0.07; 95% CI 0.26–0.12) and physical/emotional abuse (β = 0.08; 95%CI 0.2–0.04). Greater acceptance by women of positive gender norms but not -wives can disagree with partner/hold financial autonomy
Gupta et al. [40]	↓(NS)	↓	IPV C	∙∙Increased economic autonomy ∙∙Changed norms support women’s equality ∙∙Changed norms on acceptability of VAW	Addition of gender dialogue groups to group savings led to slightly lower odds (NS) of physical/sexual IPV (OR: 0.92; 95% CI: 0.58, 1.47, p = .72) but significantly reduced economic abuse 0.39 (0.25, 0.60, p = < 0.0001). Acceptance of wife beating significantly reduced, no change in attitudes towards refusing sex with partner
EMPOWERMENT + RELATIONSHIP SKILLS					
Stark Asghar 2018 [55]	No change (NC)	↓	SV + IPV PostC	∙∙Increased sense of safety in community	No significant reduction to SV (aOR = 0.96, 95% CI 0.59 to 1.57) at 12 mo/ or other violence/ perceived safety but significantly more likely to support schooling, delayed marriage/childbirth + self-reported social support systems
Stark Seff [56]	NC	↓	SV + IPV C + PostC	∙∙Increased knowledge of rights and access to services	No significant reduction to SV (OR = 0.95; 95% CI 0.65 to 1.37) or other violence; caregivers increased warmth and affection to daughters—no change to gender norms
Survivor response					
Bass et al. 2013 [43]	NA	↓	SV C	∙∙Improved survivor wellbeing/mental health	At 6 months significantly reduced PTSD (RR 5.5,95% CI: 2.5–13.2 p = < 0.001) and depression/anxiety (RR 4.6; 95% CI 2.1–11.1) compared to individual support
Vu et al. 2017 [57]	NA	↓	SV + IPV PostC	∙∙Awareness of and willingness to use services/reporting mechanisms	GBV screening and referral supports: GBV disclosure; women-centred care; GBV knowledge and services in the community; and changing norms of stigma and discrimination against the survivor
Survivor response + community mobilisation					
Hossain et al. [39]	NR	↓	SV + IPV PostC	∙∙Improved survivor wellbeing/mental health Increased sense of safety in community ∙∙Awareness of services/reporting mechanism	No significant changes were noted in perceived safety, coping, physical health, or hope. Repeat surveys indicated improved mental health but not able to be attributed to case management process. Women with the greatest psychological impacts accessed services more frequently
*Lilleston *et al*. *[54]	*NA*	*↓*	*SV* + *IPV* *PostC*	*∙∙Awareness of rights by community* *∙∙Improved survivor wellbeing/mental health*	*Reported impacts of service use included social connectedness, social opportunities, social support, strengthened family bonds, reduced distress and knowledge and skills*

Italicised rows denote qualitative studies: NA = Not applicable; NC = No change; NS = Non significant, NR = Not reported

### Study quality

The JBI quality of evidence assessment was completed for 17 of the 18 papers using tools for: i) randomised control trials (RCTs); ii) qualitative studies and; iii) cohort studies. Lack of a quality tool for case studies precluded an assessment of Bacon et al. As applied in another recent review [53], we converted scores to ratings, identifying ten studies as: Good (84–100%); six studies as Fair (50–84%); and one study as Poor (< 50%). Studies with higher quality scores tended to be cluster RCTs and RCTs while qualitative studies and mixed method studies scored lower. RCTs predominantly displayed true randomization, similar groups at baseline, reliable outcome measures consistently measured across control and intervention groups, but blinded assignment was not in most cases possible due to the nature of the interventions. Qualitative studies tended to be weaker on congruity between philosophical perspective and methodology and addressing the influence of the researcher. Overall the quality of included studies was assessed to be Fair to Good.

### Synthesis of findings

The following section synthesizes the studies by intervention type explored by level in the ecological framework from societal to individual level, as reported in Tables 3 and 4. Combined interventions are grouped according to the intervention type that appears to be the primary one.

#### Personnel

One study was identified at the societal level of intervention. Liberia’s gender sensitive police reform, aimed at increasing the proportion of female officers to 20% alongside creation of a specialist police unit for sexual violence and intimate partner violence, reported as a case study [48]. Successful recruitment occurred, however limited worker capacity building and training, and officer attrition reduced impact. Prosecution cases typically stalled in court, with a weak justice system reported to reduce effectiveness. A quality score is not available for this study, due to lack of suitable tools for case studies.

#### Community mobilization

At the community level of intervention, one paper reported on community mobilisation as a standalone intervention [45]. The intervention involved community members creating films on gender rights and respectful relationships and screening them to local audiences, to prompt dialogue. A total of 150 films were viewed by a reported 25,000 community members across the five countries (Table 3). Seventy-six interviews and 18 focus groups conducted with community members suggested positive responses to the messaging contained in the videos, with local production in local language by community members being one of the mechanisms of impact. Reported changes by participants included increased reporting of gender-based violence and community leaders taking action on forced marriage. Overall quality of the study was poor with conclusions not clearly linked to the analysis.

Community mobilisation featured as an element of two survivor response studies reported by Hossain et al. [39] and Lilleston et al. [54], further reported below. Lilleston et al.’s qualitative study, suggested that the community mobilisation element resulted in increased awareness of women’s rights among the community. Hossain et al. reported that refugee community workers engaged in activities including forums and discussions with community leaders. In neither study was community mobilisation among the interventions for which outcomes were reported, with both instead focussing on worker and service user experiences of individual interventions. Although community mobilisation is widely implemented as a stand-alone or combined intervention, there are a lack of studies evaluating its impact, perhaps reflecting the challenges and cost of comprehensively measuring impacts.

#### Social norms

Interventions aiming to change gendered social norms were explored in three cluster RCTs addressing intimate partner violence in post-conflict settings in Côte d’Ivoire, Somalia and the DRC [44, 50, 51]. All interventions involved facilitated group discussions designed to challenge community member beliefs about gendered social norms, creating expectations for gender equitable attitudes and behaviours. Trained community members delivered approximately 16 weekly sessions of discussion (duration not reported) [44, 50, 51]. The Côte d’Ivoire intervention [51] was adapted as the Engaging Men through Accountable Practice (EMAP) programme in the DRC [50]. Both provided discussion groups to men only and though both found some changes to social norms, challenges included ensuring sufficient attendance to achieve program goals. The Communities Care study in Somalia advanced social norms research, first by collaborating with community members to identify relevant norms and then testing a tool for systematically measuring social norms [44]. Delivered to mixed/single sex groups, no data were provided on attendance.

The three studies had fair [50] to good study quality [44, 51]. Lower than anticipated sample sizes limited analysis for both the Côte d’Ivoire and Somalian studies [44, 51], with only one demonstrating reductions in women’s reported physical and sexual intimate partner violence (non-significant) [51]. Overall these interventions showed promising evidence of positive changes in social norms, demonstrated through reported outcomes such as increased male involvement in household tasks, reduced male intention to perpetrate violence, and increased belief in women’s right to refuse sex, though in other respects changes regarding consent were weaker.

#### Economic empowerment

At the individual level of intervention, one RCT and a qualitative study evaluated economic empowerment, and a further two RCTs combined economic empowerment with social norms interventions. All involved micro-credit strategies, as opposed to cash transfers. Robust findings were found from all three RCTs that rated at the top end of the quality scale. Both of the economic empowerment only interventions involved livestock asset transfer. *Pigs for Peace* was offered in the DRC, with 18 month follow up finding reduced experience and perpetration of psychological abuse compared to controls [46]. Only half of the sample were partnered, leading to insufficient power for the intimate partner violence outcomes, and accordingly, non-significant results. However, significant improvement was found on subjective health, PTSD, anxiety symptoms and asset ownership (Table 4). *Solidarity Groups*, the second economic empowerment only intervention, additionally offered shared farming and group loans [47]. Like the first intervention, eligibility was not restricted to those who had experienced sexual violence/intimate partner violence to avoid stigma. Participants in this qualitative study reported economic and mental health benefits, as well a stronger sense of connection and security from working alongside other women [47].

Economic empowerment via group savings was combined with a gender norms strategy comprising eight gender dialogue groups for women and their partners, in the *EA$E* intervention in the Cote D’Ivoire [40–42]. Evaluated through an RCT comparing group savings alone to group savings with the addition of gender dialogue groups, the intervention messaged non-violence at home, respect and valuing women’s contributions to the household. Follow up 6–8 months post intervention, found economic abuse significantly reduced, but not other forms of intimate partner violence, apart from among couples attending > 75% of sessions. Mental health results from the same study [42] were mixed. Overall, women in the intervention arm were significantly less likely to meet criteria for PTSD, not extended to women experiencing intimate partner violence at baseline, though PTSD scores started from a higher base for this group. A qualitative study reporting experiences of 14 male participants, suggested that mechanisms of change for reduced economic abuse, included increased communication and joint decision making around finances [41].

Green et al. [49] also added a gender norms component to an economic empowerment program, which had previously resulted in increased controlling behaviour by partners. In the second iteration reported in the study, women were invited to include their partner/male relative to attend the training program. Women participants reported small decreases in abuse and control, and overall increases in the quality of their relationships, but less autonomy and influence over household purchases. Gibbs et al. [52] combined a monthly cash stipend with vocational, social empowerment, numeracy and savings training for women in Afghanistan. Women reported less gender inequitable attitudes, decreased food insecurity and greater involvement in household decision-making, but the intervention did not impact on intimate partner violence or depression. A sub-sample of women with baseline moderate food insecurity did however, experience reduced physical intimate partner violence, which the authors suggested may be due to greater impact of livelihood support on this group than on those with severe or nil food insecurity. Low retention may have influenced the impact of combined interventions which relied on men’s participation, with only 46% of both women and men attending 75% of EA$E sessions.

From these studies it is evident that economic empowerment programs, involving micro-credit strategies, with and without social norms strategies, have some potential to reduce abuse, and importantly improve women’s wellbeing in other ways. It is not clear that inclusion of male partners in targeted sessions accompanying economic empowerment programs is sufficient to address potential increases in economic abuse and achieving attendance by males remained elusive.

#### Empowerment

Two papers from a larger study investigated the efficacy of COMPASS—an empowerment program—to reduce girls’ experiences of violence [55, 56]. Both papers focused on conflict/ post-conflict settings in Ethiopia and the DRC respectively. COMPASS provides safe spaces, life skills and social assets, and mentors, accompanied by parallel caregiver groups. COMPASS in Ethiopia [55] consisted of 30 life skills sessions of 75 to 90 min delivered in in three refugee camps to South Sudanese girls aged 13–19 years. The intervention was complemented by 8 sessions for caregivers aimed at enhancing emotional, parental and support skills (session length not reported). In Ethiopia the focus was primarily on life skills and safe spaces, the later DRC trial enhanced the caregiver intervention [56] also providing to adolescent girls aged between 10 and 14 years 32 life skills sessions of 1–3 h duration, with caregivers in the intervention group receiving 13 sessions, substantially more than in the Ethiopian study. At 12-month follow up neither study demonstrated evidence of reduced girls’ exposure to sexual, physical or emotional violence, or transactional sex. However, in Ethiopia, significant changes were shown for girls in relation to social supports. Significant shifts were also reported in gender attitudes regarding appropriate age of marriage, birth of first child, and schooling. The DRC study found no evidence that adding a caregiver component provided a protective mechanism against sexual violence, but it did appear to increase caregiver affection towards their daughters. Study authors noted the limits to applying “best practice” parenting programs designed for high-income countries. These good quality studies suggest potential for gender norms changes among girls. High attendance by women and girls contrasts with studies involving men in gender norms programming.

#### Survivor response

Survivor response interventions included provision of care, support and protection to assist women who have experienced sexual violence and/or intimate partner violence, to recover and reduce further exposure to violence. Four studies [39, 43, 54, 57] reported on survivor responses, with two [39, 54] combining community mobilisation as reported above. Interventions focusing on survivor response strategies included a mixed methods study rated good quality on gender-based violence case management by refugee community workers [39], a CRCT rated as fair quality on group-based cognitive processing therapy [43]; a qualitative study rated as good on a mobile gender-based violence service [54]; and a mixed methods study rated as fair on screening for intimate partner violence and referral [57]. Cognitive therapy, case management and the mobile service were all reported to result in improvements in well-being, including mental health. Bass et al. [43] found significantly reduced PTSD, anxiety and depression symptoms for group-based cognitive processing therapy compared to individual support. Increased knowledge of gender-based violence, service availability and acceptability were reported by three studies [39, 54, 57]. Increases in women’s experiences of social support and connectedness were reported in the Lilleston et al. [54] study. Inclusion of community mobilisation [39, 54] appeared to strengthen the impact of survivor interventions. Use of peer refugee community workers- also survivors of gender-based violence, in the Hossain et al. Daadab study [39], improved community awareness of gender-based violence and increased acceptability of the case management service. Overall, the studies observed improved health and well-being for women, as well as increased knowledge of gender-based violence and availability and acceptability of services.

Information about the stage of the emergency in which interventions were delivered, was available for only two studies, respectively conducted in the “recovery” stage in Cote-D’Ivoire [41] and “acute” stage of the emergency in Lebanon [52].

## Discussion

This systematic review of evidence for reduced risk and incidence of sexual violence and intimate partner violence from interventions delivered in conflict, post-conflict and other humanitarian crises identified 18 papers reporting on 16 studies, all conducted in conflict/post-conflict settings in 12 countries. Four studies identified reduced incidence of intimate partner violence [40, 46, 49, 52] and one of sexual violence and intimate partner violence [51]. Although none of these studies reached statistical significance, results indicated changes in the desired direction. These studies all evaluated gendered social norms [51], or economic empowerment interventions [46] or both combined [40, 49, 52] providing the strongest evidence of impact in this review.

Some evidence of reduced *risk* factors for sexual violence/intimate partner violence was identified in all studies and intervention types against our indicators. As reported in Table 4, the most common were: changed norms related to women’s equality (5 studies); increased economic autonomy for women (5 studies) and improved survivor wellbeing/ mental health (4 studies). In addition, good evidence for improved mental health outcomes was found for economic empowerment [46] and economic empowerment plus gendered social norms interventions [42]. It should be noted that each of interventions involved micro-credit strategies, and not cash transfers, which may have different outcomes and mechanisms of impact. Secondary outcomes for reduced risk included reduced PTSD and depression/ anxiety not only for survivor support [43] but also for other types of intervention, including economic empowerment alone [46] and with gender dialogue groups [42], although not in the Gibbs study [52]. Evidence is building for social norms interventions with growing sophistication in conceptualizing and measuring change in social norms. All studies aiming to change social norms had some success, though these varied across norms and between studies and was less evident for norms relating to sexual consent. An unexpected finding was that social norms interventions predominantly focussed at the individual level with few interventions identified which targeted household/ community or societal levels. It is not clear whether this is due to the work not being undertaken or a lack of studies measuring impact. Interventions involving male partners had difficulty in impacting gender norms. Multi-strategy interventions, on the whole yielded stronger results and use of social norms interventions seems well suited to combined strategies, particularly harnessing community mobilisation.

Qualitative studies, though focussing on diverse areas, suggest experiences of social connection are an important outcome for women who participate in programming around gender-based violence [47, 52, 54], which aligns with Herman’s theory that experiences of connection are key to recovery from sexual violence/intimate partner violence [58]. Further, the qualitative studies highlight that women value hearing messaging from peers in their communities [39, 45].

In relation to interventions delivered in conflict vs post-conflict settings—no trend is evident in relation to intervention or study type or outcomes. We identified no studies which met inclusion criteria, undertaken in disasters or epidemics. There was a dearth of studies evaluating community led initiatives, instead most interventions included in this review were delivered by international NGOs. This may reflect a lack of evaluation of such interventions or simply an absence in the peer reviewed literature, signalling at least, the need for local partnerships to ensure adapted or context sensitive interventions that draw further on existing local knowledge.

Over the past ten years, there has been a decrease in studies evaluating survivor responses to sexual violence in conflict and an increase in studies on primary prevention, particularly of intimate partner violence in post-conflict settings, including considerable work with men. Of the 16 studies, six targeted men as participants either exclusively [50, 51]; as a specific focus of a component of the intervention [41, 45, 49]; or as part of interventions targeting community members more broadly [44, 46].

In contrast to our earlier review [31], we found no studies where risk had appeared to *increase* as a result of interventions. Conversely, it is obvious that in many studies careful attention has been paid to inclusive approaches to delivering interventions in order to: attend to safety and avoid stigmatising/identifying women who have or continue to experience abuse; and to maximising transparency to communities. This contributed to under-powering of studies, a feature of multiple studies in this review—which needs to be recognised as a necessary cost to safe programming and research. Overall, it is clear that while a range of gender-based violence prevention programming is currently being implemented in conflict/post-conflict settings, evidence for the effectiveness of interventions for prevention remains limited. This aligns with two systematic reviews which explored interventions to address gender-based violence specifically in refugee settings [4, 34]. The What Works synthesis brief on evidence to prevent violence against women and girls in conflict and humanitarian crisis also found insufficient evidence to classify any intervention as “effective,” but found positive results from community-based programming focussing on social norms change, and mixed results from economic empowerment interventions [59].

The heterogeneity of interventions, contexts, study types and findings constrained the degree of synthesis possible from this review. However, some overall patterns emerge from our analysis. Firstly, it is clear that demonstrating robust reduction in sexual violence and/or intimate partner violence as a result of interventions may remain elusive in humanitarian settings. A factor in this situation may be the efforts by researchers to undertake transparent and non-stigmatising delivery of interventions and study methods, which have been rightly privileged. Secondly, improvements to mental health, social support and other positive outcomes are clear, even where reduced abuse cannot be established, particularly in relation to social norms and some economic empowerment interventions. Similar outcomes have been found from interventions in non-humanitarian crisis settings, with reduced abuse a harder goal to achieve [60, 61]. Thirdly, programmes addressing structural issues cannot only focus on women, yet have found men challenging to engage. Fourthly, most programming was relatively short term with no follow-up periods longer than two years. These attributes reflect finite resources, however changes to patterns of abuse and gendered social norms are long term outcomes which require longer time frames to accurately measure impact. Fifthly, the studies in this review show a strong emphasis on intimate partner violence or combined intimate partner violence and sexual violence, leaving an ongoing evidence gap in relation to prevention of sexual violence, which remains a challenge in all settings, not just humanitarian contexts. Sexual violence interventions in conflict settings, including non-partner sexual violence appear to have diminished as a focus of research in the past ten years, based on studies identified for this review. The diverse methodologies applied in identified studies are a strength of the field, in particular the use of RCT and CRCTs which provide robust opportunities for comparison, as well as qualitative studies which have capacity for rich insights, to foreground the lived experience of participants receiving interventions, though among studies in this review, these were not always clearly reported. Finally, compared to our earlier review there is a greater volume of interventions targeting structural/ community level change than individual level change. In part, this reflects the strong interest in social norms interventions.

### Limitations

Studies that did not explicitly refer to violence/abuse, or to conflict/post-conflict/crisis in the key words, title or abstract may not have been identified. The search also excluded studies with titles, abstracts or full-texts in languages other than English. The disparate interventions allowed for limited synthesis, as did the disparate target groups and study designs. For example among studies without control groups it is not possible to establish if observed changes were due to the intervention. Limited data were available on most changes to risk, and our adopted indicators may have over-rated reductions in risk. There was a lack of findings in relation to the diversity of participants in studies, and as such an intersectional lens to results has not been applied. Lastly, this study focussed only on interventions delivered in low- and middle-income countries, reflecting the unique challenges they face during conflict and other humanitarian crises.

## Conclusion

From a policy and practice perspective interventions for changing social norms on gender equality/ acceptability of violence against women, appear to hold promise, including as an adjunct to economic empowerment, although men’s programming for gendered norms needs to address participation rates. It is not yet clear how combining social norms with economic empowerment interventions can best address potential increases in economic abuse and more research is needed on this. The findings also point to the need to work locally with partners to ensure that programs are contextually adapted and privilege community-generated interventions. Methodological strengths of Future research should continue to explore how social norms change interventions can be most effectively delivered, including the impact of having mixed and/or same sex groups. Significant reductions in sexual violence/intimate partner violence incidence is unusual as an outcome from RCTs in all settings, but particularly hard to achieve in humanitarian settings which suggests a need for outcome measures that reflect these realities and recognition that null results are not indicative of no impacts. Other research implications are the need for well-executed qualitative research to understand participants’ experiences of interventions and the mechanisms of change, including from RCTs. This review indicates that significant effort has been directed towards building the evidence base for interventions addressing sexual and intimate partner violence, although with a larger focus on intimate partner violence. While there has been some progress and promising results, there is more work to do to address both sexual violence and intimate partner violence.

## Supplementary Information

**Additional file 1**. Indicators for reduced risk and incidence of sexual and intimate partner violence.

## Data Availability

The datasets used in the current study are available from the corresponding author on reasonable request.

## Abbreviations

C: Conflict
DRC: Democratic Republic of Congo
EE: Economic empowerment
GBV: Gender-based violence
IPV: Intimate partner violence
NGO: Non-government organization
PC: Post-conflict
RCT: Randomized control trial
SV: Sexual violence
WHO: World Health Organization
