# Supplementary material for: The impact of interventions to reduce risk and incidence of intimate partner violence and sexual violence in conflict and post-conflict states and other humanitarian crises in low and middle income countries: a systematic review
Source: Confl Health. 2021 Nov 24;15:86. doi: 10.1186/s13031-021-00417-x (PMC8611888; doi:10.1186/s13031-021-00417-x)
Supplement: Supplementary file 1 — Additional file 1. Indicators for reduced risk and incidence of sexual and intimate partner violence. [file 13031_2021_417_MOESM1_ESM.docx]

**Additional file 1**

**Indicators for reduced risk and incidence of sexual and intimate partner violence**

- Increased sense of safety in community
- Increased sense of safety in the home
- Intervention with survivors results in improved wellbeing / mental health
- Reintegration/livelihood programs to survivors reduces exposure to sexual violence (SV) /enhances possibilities of leaving an abusive situation
- Combat leaders engaged to halt SV
- Disarmament, demobilisation and reintegration (DDR) programs implemented
- DDR programs include safety/livelihood programs for women/girls
- Women in peace-building targeting SV and intimate partner violence (IPV)
- Awareness of rights by community
- Awareness of availability of services/reporting mechanisms
- Willingness to use reporting mechanisms and uptake of services
- Implementation/impact of codes of conduct/training
- Gender specific (ie female) recruitment implemented
- Minimum female representation in negotiating/ local representative bodies
- Disciplinary action initiated
- Coordination mechanisms established
- Impact of patrols to increase women’s safety
- Alternatives provided to firewood/water collection
- Completion of situational analysis of risk of SV
- Impacts of infrastructure designed for risk reduction eg lighting/ water, sanitation and hygiene (WASH) facilities
- Systems for distribution of food/other resources established for reduced sexual exploitation and abuse
- Legal action initiated/convictions
- Country action on International Criminal Court provisions
- Women report living a life free of violence
- Programmes to address violence against women (VAW) are widely implemented
- Increased resources and political will to address VAW
- Increased awareness about VAW as a public health and development problem and that it is preventable
- Sectoral outcomes related to health, economic, and social development improved (e.g. improved mental health, reduced household poverty, improved women’s and child health, improved women’s education and earnings, and reduced absenteeism)
- Increased access to legal recourse and reduction of impunity
- Families, communities and institutions believe in and uphold gender equality as a norm and no longer accept VAW
- Men accept and treat women as equals
- Women can make autonomous decisions
- Women have knowledge of their rights and access to programmes
- Household poverty reduced
- Increased economic autonomy for women
- Changed norms supporting women’s equality
- Changed norms on acceptability of VAW
- Men show reduced acceptance of VAW
